# Anemia in Pregnancy: Incidence, Risk Factors, and Outcomes

**DOI:** 10.7759/cureus.93807

**Published:** 2025-10-04

**Authors:** Lateefa O Aldakhil, Saleh Alobaid, Adnan T Almogbel, Saad M Alfouzan

**Affiliations:** 1 Department of Obstetrics and Gynecology, King Saud University Medical City, King Saud University, Riyadh, SAU; 2 Department of Internal Medicine, King Saud University Medical City, King Saud University, Riyadh, SAU; 3 Department of Internal Medicine, King Fahad Medical City, Riyadh, SAU

**Keywords:** anemia in pregnancy, antenatal care, iron deficiency anemia, pregnancy outcomes, risk factors

## Abstract

Background: In Saudi Arabia, the reported prevalence of anemia in pregnancy varies considerably, reflecting differences in sociodemographic characteristics and nutritional status across study populations.

Objectives: This study aimed to (i) determine the prevalence of anemia at the first antenatal visit; (ii) assess prevalence across pregnancy trimesters; (iii) identify risk factors, including compliance with iron and multivitamin supplementation; and (iv) evaluate maternal and neonatal outcomes associated with anemia.

Methods: We conducted a prospective cohort study at the antenatal clinic of King Khalid University Hospital, Riyadh, Saudi Arabia, between January 2020 and December 2022. Pregnant women attending their first antenatal visit were consecutively recruited and followed throughout pregnancy. Hemoglobin (Hb) was measured in each trimester, and anemia was defined according to the World Health Organization (WHO) criteria. Outcomes included postpartum hemorrhage (PPH, transfusion, preterm birth, low birth weight (LBW), and neonatal intensive care unit (NICU) admission. Notably, Hb data were available for 220 women (21.9%) in the first trimester, primarily due to late booking. This limitation was acknowledged to ensure transparency regarding potential selection bias.

Results: A total of 1003 women were included. The prevalence of anemia increased with gestational age: 26 women (11.8%) at the first visit, 105 women (14.4%) in the second trimester, and 289 women (28.8%) in the third trimester. Advancing gestational age was an independent predictor of anemia (adjusted OR 1.03, 95% CI 1.01-1.05, p = 0.007). Anemia was significantly associated with PPH (18 women (6.3%) vs. 23 women (3.6%), p = 0.047), but not with LBW or NICU admission. Iron supplementation increased progressively during pregnancy, but postpartum continuation was significantly lower among anemic women compared with non-anemic women (195 (67.5%) vs. 528 (83.4%), p = 0.035).

Conclusion: Anemia in pregnancy is common among Saudi women and is associated with maternal complications and suboptimal adherence to supplementation. Early screening, strategies to improve compliance, and culturally tailored nutritional counseling are essential to reduce risks.

## Introduction

Anemia in pregnancy remains one of the most common global health challenges, affecting approximately 40% of pregnant women worldwide and contributing significantly to maternal and perinatal morbidity and mortality [1-5]. The World Health Organization (WHO) defines anemia in pregnancy as a hemoglobin (Hb) concentration <110 g/L during pregnancy, with severity classified as mild (100-109 g/L), moderate (70-99 g/L), and severe (<70 g/L) [1]. This condition compromises oxygen delivery, leading to maternal fatigue, reduced work capacity, and heightened susceptibility to infections [2,6,7], while also posing risks to fetal neurodevelopment through impaired cerebral energy metabolism [8].

Hemorrhage and anemia remain major contributors to maternal mortality. Between 2003 and 2009, hemorrhage accounted for 27% of maternal deaths globally, with nearly two-thirds attributable to postpartum hemorrhage (PPH) [9]. Anemia further exacerbates this risk by limiting compensatory capacity during obstetric bleeding and increasing susceptibility to sepsis and heart failure [3,4]. Recent estimates indicate that anemia, particularly iron-deficiency anemia (IDA), accounts for approximately 20%-22% of maternal deaths in low- and middle-income countries [3,4]. Severe anemia (Hb < 60-80 g/L) has been consistently associated with adverse maternal outcomes, including intensive care unit (ICU) admission and increased peripartum mortality, as well as poor perinatal outcomes such as preterm delivery, low birth weight (LBW), stillbirth, and neonatal mortality [10-14].

Pregnancy imposes substantial hematologic demands, with physiological hemodilution arising from a disproportionate increase in plasma volume relative to red cell mass, peaking at around 32 weeks of gestation [15,16]. The total iron requirement during pregnancy is estimated at ~840 mg, yet fewer than 20% of women worldwide begin pregnancy with iron stores exceeding 500 mg [17]. Consequently, iron deficiency is the predominant cause of anemia in pregnancy, responsible for ~50% of cases globally [18]. Additional contributors include folate and vitamin B12 deficiencies, hemoglobinopathies, malaria, and chronic disease [2,19]. While mild anemia may not consistently affect maternal or neonatal outcomes in high-resource settings [20-22], moderate to severe anemia is clearly associated with adverse consequences across diverse populations [12-14].

Iron supplementation remains central to the prevention and treatment of anemia in pregnancy. A Cochrane review confirmed that daily iron supplementation improves Hb concentrations and reduces the risk of anemia [23]. Randomized trials also suggest benefits for birth weight, even among iron-replete women [24]. However, the role of routine supplementation in non-anemic populations remains debated, given concerns about oxidative stress, pre-eclampsia, and gestational diabetes associated with high iron stores [25-27]. Accurate assessment of iron status is also challenging: Hb levels decline physiologically in late pregnancy, while ferritin is influenced by inflammation [27]. These complexities underpin variations in global guidelines. The WHO recommends routine daily iron (30-60 mg) in regions where anemia prevalence exceeds 40%, and intermittent dosing in lower-prevalence areas [28]. By contrast, high-income countries such as Canada, the UK, Australia, and the US generally favor targeted supplementation, with optional low-dose prophylaxis [29-33]. In Saudi Arabia, reported prevalence estimates vary by region and trimester, ranging from 19% to over 40%, with higher rates documented in high-altitude areas [34-36]. Within the Eastern Mediterranean Region, anemia remains highly prevalent, affecting 69% of pregnant women [31]. Local studies in Saudi Arabia highlight multifactorial contributors, including inadequate dietary intake, poor adherence to iron supplementation [37], grand multiparity, short interpregnancy intervals [38,39], and sociodemographic factors [34-36,38]. Moreover, maternal mortality surveys from Saudi tertiary centers underscore anemia and hemorrhage as leading contributors to adverse maternal outcomes [40]. However, most available evidence is derived from cross-sectional or retrospective designs, with limited prospective data directly linking anemia to maternal and neonatal outcomes in the Saudi context.

The present prospective cohort study was designed to (i) determine the prevalence of anemia at the first antenatal visit; (ii) assess its prevalence across pregnancy trimesters; (iii) identify risk factors, including nutritional intake, adherence to iron supplementation, and reproductive history; and (iv) examine maternal and neonatal outcomes associated with anemia, such as preterm birth, low birth weight, postpartum hemorrhage, and Apgar scores. By generating prospective, locally relevant data, this study seeks to address existing knowledge gaps and provide evidence to strengthen antenatal care strategies in Saudi Arabia.

## Materials and methods

This prospective cohort study was conducted at the antenatal clinic of King Khalid University Hospital, King Saud University Medical City, Riyadh, Saudi Arabia, between January 2020 and December 2022. The hospital is a tertiary academic referral center that provides comprehensive obstetric services, including a dedicated low-risk obstetric clinic, where the study was based. All pregnant women attending their first antenatal visit at the low-risk clinic during the study period were eligible, consecutively recruited, and followed throughout pregnancy. 

Exclusion criteria included women with known hemoglobinopathies (e.g., sickle cell disease, thalassemia), multiple pregnancies, or a history of infertility treatment. Additional exclusions were chronic conditions commonly associated with anemia, such as chronic kidney disease, chronic infection, autoimmune disorders, and a history of gastric sleeve surgery. These criteria ensured that the study population represented women with low-risk singleton pregnancies (Table 1).

**Table 1:** Inclusion and exclusion criteria. KKUH: King Khalid University Hospital.

Criteria	Details
Inclusion	Pregnant women attending their first antenatal visit at the low-risk clinic at KKUH outpatient setting. Singleton pregnancy.
Exclusion	Known hemoglobinopathies (e.g., sickle cell disease, thalassemia). Multiple gestations. Chronic illnesses commonly associated with anemia, such as chronic renal disease, chronic infection, and autoimmune diseases. Post-sleeve gastrectomy. History of infertility treatment.

At recruitment (first antenatal visit), demographic and clinical data were obtained, including age, parity, gravidity, interpregnancy interval, and BMI. Nutritional and supplement intake history (iron and multivitamins) was recorded. Women were followed prospectively at each trimester, with data collected on compliance with prescribed supplementation, development of anemia, and pregnancy outcomes. Hb concentration was measured at the first visit and at subsequent trimester visits, using an automated hematology analyzer in the hospital laboratory, standardized to international quality control protocols. Anemia was defined according to the WHO criteria: Hb < 110 g/L in pregnancy, classified as mild (100-109 g/L), moderate (70-99 g/L), and severe (<70 g/L) [1]. Serum ferritin levels were obtained in a subset of patients when clinically indicated to evaluate iron stores. Notably, Hb data were available for only 220 women (21.9%) in the first trimester, primarily due to late booking and incomplete early laboratory documentation. Missing data were handled using an available-case analysis (complete case analysis), whereby women with missing values were excluded from the specific analysis but retained in all other analyses where data were available.

The primary outcome was the prevalence of anemia across pregnancy trimesters. Secondary outcomes included maternal outcomes such as postpartum hemorrhage (PPH), transfusion, or ICU admission. Neonatal outcomes included preterm birth (<37 weeks), LBW (<2.5 kg), Apgar scores at one and five minutes, and NICU admission. Compliance outcomes were adherence to prescribed iron and multivitamin supplementation, assessed by patient self-report at follow-up visits.

Data were entered and analyzed using IBM SPSS Statistics for Windows, Version 23.0 (Released 2015; IBM Corp., Armonk, NY, US). Categorical variables were summarized as frequencies and percentages, and continuous variables as means ± standard deviations (SD) or medians (interquartile ranges, IQR), depending on distribution. The prevalence of anemia was calculated for each trimester. Associations between anemia and categorical risk factors were assessed using the chi-square (χ²) or Fisher’s exact test, while continuous variables were compared using the Student’s t-test or Mann-Whitney U test. Logistic regression was used to identify independent predictors of anemia and adverse outcomes, adjusting for potential confounders. A p-value < 0.05 was considered statistically significant.

For categorical comparisons, we used Pearson’s chi-square test, reporting the chi-square statistic (χ²), degrees of freedom (df), p-value, and effect size (Cramér’s V; 0.10/0.30/0.50 ≈ small/medium/large). Odds ratios (anemic vs non-anemic) with 95% CIs were additionally reported for 2 × 2 comparisons. Fisher’s exact test was used when expected cell counts were <5 (e.g., stillbirth). All tests were two-sided, with α = 0.05.

## Results

A total of 1003 pregnant women were included. The majority of women had their first antenatal visit in the second or third trimester. The median maternal age was 31 years (IQR 27-36), and the median gestational age at recruitment was 16 weeks. Most participants were multigravida (n = 786, 78.4%) with a median gravidity of 3, and multipara (n = 570, 56.8%) with a median parity of 2. Nearly half (49.2%) reported previous contraceptive use, and one-third (n = 316, 31.5%) reported planned pregnancies. The majority (n = 707, 70.5%) were receiving folic acid supplementation. Hypothyroidism (n = 79, 7.9%) was the most common chronic condition, followed by asthma (n = 41, 4.1%) and diabetes mellitus (n = 25, 2.5%) (Table 2).

**Table 2:** Baseline demographic and clinical characteristics of the study population (n = 1003). GA: gestational age.

Variable	Number	Percentage
Age (years)		
Median (IQR)	31 (27-36)	31% (27-36)
Past medical history		
No	760	75.8%
Asthma	41	4.1%
Diabetes mellitus	25	2.5%
Hypertension	13	1.3%
Hypothyroidism	79	7.9%
Polycystic ovary syndrome	8	0.8%
Others	80	8.0%
N/A	4	0.4%
Gravidity		
Primigravida	217	21.6%
Multigravida	786	78.4%
Median (IQR)	3 (2-4)	3% (2-4)
Parity		
Nullipara	215	21.4%
Primipara	218	21.7%
Multipara	570	56.8%
Median (IQR)	2 (1-3)	2% (1-3)
GA (weeks)		
Median (IQR)	16 (11-21)	16% (11-21)
Contraception history		
No	508	50.6%
Yes	493	49.2%
N/A	2	0.2%
Planned pregnancy		
No	684	68.2%
Yes	316	31.5%
N/A	3	0.3%
Folic acid intake		
No	293	29.2%
Yes	707	70.5%
N/A	3	0.3%
Duration (months), median (IQR)	3 (3-4)	3% (3-4)
Dose (mg), median (IQR)	1 (1-2)	1% (1-2)

At the first trimester, Hb data were available for only 220 women (21.9%), primarily due to late booking. The median Hb was 12.4 g/dL, and anemia (Hb < 11 g/dL) was present in 26 women (11.8%). In the second trimester, Hb data were available in 728 women (72.6%), with a median Hb of 11.6 g/dL. Anemia prevalence was 14.4% at this stage (Hb < 10.5 g/dL). By the third trimester, Hb data were available for 921 women (91.8%), with a median Hb of 11.5 g/dL. The prevalence of anemia rose markedly to 28.8% (n = 289). Postpartum analysis showed that 12.5% (n = 115) of women were anemic (Hb < 10 g/dL) (Table 3). Overall, there was a significant association between gestational age and anemia prevalence (p = 0.015).

**Table 3:** Anemia prevalence by trimester and postpartum. Anemia in pregnancy was defined according to the WHO thresholds: hemoglobin (Hb) < 11 g/dL in the first and third trimesters, and <10.5 g/dL in the second trimester.

Stage of pregnancy	Hb data available (n, %)	Median Hb (g/dL)	Anemia cases (n, %)
First trimester	220 (21.9%)	12.4	26 (2.6%)
Second trimester	728 (72.6%)	11.6	105 (10.5%)
Third trimester	921 (91.8%)	11.5	265 (28.8%)
Postpartum	922 (92 %)	10.9	115 (12.2%)

Comparison of maternal characteristics between anemic (n = 289) and non-anemic women (n = 633) in the third trimester (Table 4) showed no significant differences in age, gravidity, parity, contraception history, pregnancy planning, or folic acid intake. Asthma was less common among anemic women (1.4% vs. 4.6%; χ²(1, N = 922) = 4.99; p = 0.026; Cramér’s V = 0.07 (small effect)). No significant associations were found for diabetes (χ²(1) = 0.00, p = 1.00), hypertension (χ²(1) = 0.00, p = 1.00), hypothyroidism (χ²(1) = 0.54, p = 0.463), gravidity (χ²(1) = 2.84, p = 0.092), parity (χ²(2) = 4.11, p = 0.128), contraception history (χ²(1) = 0.02, p = 0.894), planned pregnancy (χ²(1) = 0.00, p = 1.00), or folic acid intake (χ²(1) = 0.49, p = 0.482). Effect sizes (Cramér’s V) were consistently small (<0.10).

**Table 4:** Association between maternal characteristics and anemia status at the third trimester.

Variable	Non-anemic (n = 633)	Anemic (n = 289)	p-value
Age (years)			
Median (IQR)	31 (27-35)	31 (27-36)	0.869
Past medical history			
No	480 (75.8%)	227 (78.5%)	0.655
Yes	151 (23.9%)	61 (21.1%)	0.655
Asthma	29 (4.6%)	4 (1.4%)	0.053
Diabetes mellitus	16 (2.5%)	8 (2.8%)	0.975
Hypertension	8 (1.3%)	3 (1%)	0.956
Hypothyroidism	52 (8.2%)	19 (6.6%)	0.686
Polycystic ovary syndrome	4 (0.6%)	3 (1%)	0.802
Others	47 (7.4%)	25 (8.7%)	0.81
Gravidity			
Primigravida	149 (23.5%)	53 (18.3%)	0.077
Multigravida	484 (76.5%)	236 (81.7%)	0.077
Median (IQR)	3 (2-4)	3 (2-4)	0.225
Parity			
Nullipara	149 (23.5%)	51 (17.6%)	0.128
Primipara	134 (21.2%)	64 (22.1%)	0.128
Multipara	350 (55.3%)	174 (60.2%)	0.128
Median (IQR)	2 (1-3)	2 (1-3)	0.169
Gestational age at booking, median (IQR)	15 (11-20)	17 (11-23)	0.015
Contraception history	313 (49.4%)	145 (50.2%)	0.625
Planned pregnancy	198 (31.3%)	91 (31.5%)	0.995
Folic acid intake	454 (71.7%)	200 (69.2%)	0.737
Duration (months), median (IQR)	3 (3-4)	3 (3-4)	0.666
Dose, median (IQR)	1 (1-2)	1 (1-2)	0.884

When stratified by anemia status, iron supplementation was significantly higher among anemic women compared with non-anemic women (Table 5). In the second trimester, 81.6% of anemic women used iron versus 57.4% of non-anemic women (χ²(1, N = 728) = 21.81; p < 0.001; V = 0.17 (medium effect)). In the third trimester, iron intake remained higher in anemic women (90.2% vs. 72.0%; χ²(1, N = 921) = 35.99; p < 0.001; V = 0.20 (medium effect)). However, postpartum iron use was significantly lower among anemic women (67.5% vs. 83.4%; χ²(1, N = 922) = 28.85; p < 0.001; V = 0.18 (medium effect)). These findings suggest that continued postpartum iron use is protective against anemia.

**Table 5:** Association between iron supplementation and anemia status during pregnancy and postpartum.

Stage of pregnancy	Iron supplementation in anemic women, n (%)	Iron supplementation in non-anemic women (%)	p-value
Second trimester	86 (81.6%)	357 (57.4%)	<0.001
Third trimester	260 (90.2%)	454 (72.0%)	<0.001
Postpartum	195 (67.5%)	528 (83.4%)	0.035

Pregnancy outcomes were broadly similar between groups (Table 6). Full-term delivery occurred in 85% of non-anemic vs. 81% of anemic women (χ²(1, N = 1003) = 1.60; p = 0.205; V = 0.04). Preterm birth rates were 7.4% vs. 10% (χ²(1) = 1.60, p = 0.205, V = 0.04). Abortions occurred in 7.6% vs. 9.2% (χ²(1) = 0.58, p = 0.445, V = 0.02). Stillbirth was rare and not significant (χ²(1) = 0.00, p = 1.00). PPH was more frequent in anemic women (6.3% vs. 3.6%; χ²(1, N = 1003) = 2.52; p = 0.112; V = 0.05 (small)). Blood transfusion (2.8% vs. 1.6%; χ²(1) = 0.90; p = 0.344; V = 0.03) and mode of delivery (vaginal, instrumental, cesarean; χ²(2, N = 1003) = 3.75; p = 0.153; V = 0.06) showed no significant associations. Neonatal outcomes were also similar: median birth weight (3.08 vs. 3.07 kg, p = 0.895), NICU admission (8.7% vs. 6.0%; χ²(1) = 1.75, p = 0.186, V = 0.04), and neonatal complications (8.3% vs. 6.3%; χ²(1) = 0.90, p = 0.344, V = 0.03).

**Table 6:** Maternal and neonatal outcomes in relation to antenatal anemia status.

Variable	Non-anemic (n = 688)	Anemic (n = 315)	p-value
Pregnancy outcome			
Full term	585 (85%)	255 (81.3%)	0.422
Pre-term	51 (7.4%)	31 (10%)	0.422
Abortion	52 (7.6%)	29 (9%)	0.384
Stillbirth	1 (0.2%)	0 (0%)	0.422
Postpartum hemorrhage	23 (3.6%)	18 (6.3%)	0.047
Blood transfusion	10 (1.6%)	8 (2.8%)	0.143
Type of delivery			
Vaginal	329 (48.6%)	127 (41.2%)	0.143
Instrumental	85 (12.6%)	39 (12.7%)	0.143
Cesarean section	225 (33.2%)	117 (38%)	0.143
Newborn weight (kg)			
Median (IQR)	3.07 (2.76-3.36)	3.08 (2.7-3.41)	0.895
NICU admission	38 (6%)	25 (8.7%)	0.266
Complication	40 (6.3%)	24 (8.3%)	0.478

Univariate analysis identified asthma (protective, OR 0.29, p = 0.022) and higher gestational age (OR 1.03, p = 0.005) as significantly associated with anemia. Multivariable logistic regression confirmed gestational age as an independent predictor of anemia (adjusted OR 1.03, 95% CI 1.01-1.05, p = 0.007) (Table 7).

**Table 7:** Logistic regression analysis of predictors of anemia in pregnancy.

Variable	Univariate analysis	Univariate analysis	Univariate analysis	Univariate analysis	Multivariate analysis	Multivariate analysis	Multivariate analysis
Variable	Unadjusted OR	95% CI	p-value	Adjusted OR	Adjusted OR	95% CI	p-value
Age (years)	1.001	0.98-1.03	0.967				
Past medical history							
Asthma	0.29	0.1-0.84	0.022	0.34	0.34	0.12-1	0.05
Diabetes mellitus	1.1	0.46-2.6	0.831				
Hypertension	0.82	0.22-3.11	0.77				
Hypothyroidism	0.79	0.46-1.36	0.388				
Polycystic ovary syndrome	1.65	0.37-7.42	0.514				
Others	1.18	0.71-1.96	0.519				
Gravidity	1.03	0.97-1.09	0.37				
Parity	1.04	0.98-1.11	0.209				
Gestational age	1.03	1.01-1.04	0.005	1.03	1.03	1.01-1.05	0.007
Contraception history	1.02	0.77-1.35	0.873				
Planned pregnancy	1.01	0.75-1.36	0.947				
Folic acid intake	0.89	0.65-1.2	0.437				
Duration (months)	0.99	0.92-1.05	0.682				
Dose (mg)	0.995	0.9-1.1	0.929				

Iron supplementation increased progressively during pregnancy. In the first trimester, only 24 women (11%) reported use, compared with 433 women (59.5%) in the second trimester and 694 women (75.4%) in the third trimester. A similar pattern was observed with multivitamin intake: 17 women (7.8%) in the first trimester, 449 women (61.6%) in the second trimester, and 773 women (84.4%) in the third trimester. These findings demonstrate that adherence to supplementation improved as pregnancy advanced, although uptake during the early stages remained relatively low.

## Discussion

This prospective cohort study demonstrates that anemia in pregnancy remains a significant public health problem, with prevalence rising from 11.8% (n = 26) in the first trimester to 28.8% (n = 289) in the third trimester (Table 3). This pattern reflects both the physiological hemodilution of pregnancy and the inadequacy of iron intake to meet gestational demands. Globally, the WHO estimates that anemia affects nearly 40% of pregnant women [1,2], and similar prevalence rates have been reported in studies from Saudi Arabia [32,33,35].

No significant associations were found between anemia and maternal age, parity, gravidity, contraceptive use, or chronic conditions (Table 4). However, gestational age was identified as an independent predictor of anemia in multivariable regression (Table 5), consistent with systematic reviews showing that Hb concentration typically declines as pregnancy advances [16,22]. Interestingly, asthma appeared protective in univariate analysis, though this finding should be interpreted cautiously due to the small subgroup size.

Iron supplementation emerged as a key determinant of anemia status. Anemic women reported higher iron use in the second and third trimesters (Table 5), reflecting increased prescription following diagnosis. However, in the postpartum period, continuation rates of iron were significantly lower among anemic women compared with non-anemic women (67.5% (n = 195) vs. 83.4% (n = 528), p = 0.035), indicating poor long-term compliance. This is in line with earlier Saudi and regional studies highlighting barriers such as gastrointestinal side effects, limited awareness, and cultural influences on adherence [37]. Importantly, our findings suggest that continued postpartum iron supplementation is protective against persistent anemia, supporting WHO recommendations for extending supplementation beyond delivery in high-prevalence settings [18,23]. WHO guidelines recommend routine folic acid and iron supplementation for all pregnant women, regardless of Hb status [23]. In contrast, supplementation in our cohort was largely reactive, initiated after the diagnosis of anemia. This explains the higher proportion of anemic women among those reporting iron use, a finding most likely attributable to reverse causation, and underscores a gap between international recommendations and local practice. Nevertheless, all participants received standard antenatal care in accordance with hospital and national guidelines, ensuring no ethical concerns regarding treatment withholding.

Anemia was significantly associated with PPH (6.3% (n = 18) vs. 3.6% (n = 23), p = 0.047) (Table 6), consistent with evidence that maternal anemia increases the risk of adverse maternal outcomes including obstetric hemorrhage and intensive care admission [2,9,10]. Although no statistically significant associations were found with preterm birth, LBW, or NICU admission, the observed trends align with meta-analyses showing maternal anemia as an independent risk factor for preterm delivery and LBW [12,14,41,42]. The clinical importance of even mild anemia in our cohort is noteworthy, as in resource-constrained contexts with limited nutritional reserves, small reductions in Hb may contribute to meaningful increases in maternal and perinatal risks [4,13]. The strengths of this study include its prospective design, large sample size, and comprehensive assessment of maternal and neonatal outcomes, which increase the reliability and applicability of findings to clinical practice. The limitations include reliance on Hb as the sole marker of anemia, without routine ferritin or micronutrient assays to differentiate iron deficiency from other causes; reliance on self-reported supplementation adherence, which is prone to recall bias; and the single-center design, which may limit generalizability to other Saudi regions with different sociodemographic profiles. Despite these limitations, the study provides valuable prospective evidence from Saudi Arabia, reinforcing the need for early and universal screening, proactive supplementation strategies, and culturally tailored nutritional counseling. From a policy perspective, these findings are aligned with the WHO Global Nutrition Targets for 2025, which call for a 50% reduction in anemia among women of reproductive age [17]. Locally adapted programs addressing biomedical, dietary, and sociocultural factors are urgently needed to improve maternal and neonatal outcomes.

This study has several notable strengths. Its prospective cohort design, large sample size, and systematic follow-up across all trimesters provided robust estimates of anemia prevalence and enabled assessment of maternal and neonatal outcomes. The use of standardized laboratory methods and consistent definitions enhanced the reliability of findings.

Nevertheless, some limitations should be acknowledged. Baseline values were incomplete Hb values, as the majority of women initiated antenatal care in the second or third trimester, which limited the accuracy of early prevalence estimates and may have introduced selection bias. In addition, serum ferritin was measured only when clinically indicated, which restricted our ability to differentiate iron deficiency from IDA. Finally, supplementation adherence was self-reported and subject to recall bias.

Despite these limitations, this study provides valuable prospective evidence from a Saudi cohort and highlights important opportunities for earlier screening, improved supplementation strategies, and culturally tailored interventions.

## Conclusions

Anemia in pregnancy remains a prevalent and clinically significant condition among Saudi women, with the prevalence increasing progressively across gestation. Our findings highlight that advancing gestational age, poor compliance with supplementation, and limited postpartum continuation of iron therapy are key contributors. Anemia was significantly associated with maternal complications, particularly postpartum hemorrhage, and showed trends toward adverse neonatal outcomes. These results emphasize the importance of early and universal screening at the first antenatal visit, adherence to iron and multivitamin supplementation throughout pregnancy and postpartum, and culturally tailored nutrition counseling. Strengthening patient education and addressing barriers to compliance are critical steps.
